# Migraine screen questionnaire: further psychometric evidence from categorical data methods

**DOI:** 10.1186/s12955-020-01361-9

**Published:** 2020-04-28

**Authors:** Md. Dilshad Manzar, Unaise Abdul Hameed, Mohammed Salahuddin, Mohammad Yunus Ali Khan, Dejen Nureye, Wakuma Wakene, Majed Alamri, Abdulrhman Albougami, Seithikuruppu R. PandiPerumal, Ahmed S. Bahammam

**Affiliations:** 1 Department of Nursing, College of Applied Medical Sciences, Majmaah University, Majmaah, 11952 Saudi Arabia; 2 Department of Physiotherapy, Fatima College of Health Sciences, Al Mafraq, Abu Dhabi City, UAE; 3 Department of Pharmacy, College of Medicine and Health Sciences, Mizan-Tepi University (Mizan Campus), Mizan-Aman, Ethiopia; 4 Pharmacology Division, Department of BioMolecular Sciences, University of Mississippi, Oxford, USA; 5 Department of Biomedical Sciences, College of Medicine and Health Sciences, Mizan-Tepi University (Mizan Campus), Mizan-Aman, Ethiopia; 6 Department of Anatomy, Arabian Gulf University, Manama, Bahrain; 7 Department of Clinical Pharmacy, Mettu University, Mettu, Ethiopia; 8 Nursing Department, College of Applied Medical Sciences, University of Hafr Al Batin, Haf Al Batin, Saudi Arabia; 9 Somnogen Canada Inc, College Street, Toronto, ON Canada; 10 The University Sleep Disorders Center, College of Medicine, King Saud University, Box 225503, Riyadh, 11324 Saudi Arabia; 11 National Plan for Science and Technology, College of Medicine, King Saud University, Riyadh, Saudi Arabia

**Keywords:** Headache, Student, Africa, Factor analysis, McDonald’s omega

## Abstract

**Background:**

Psychometric investigations of tools used in the screening of migraine including the migraine screen questionnaire (MS-Q), using an adequate statistical approach is needed. We assessed the psychometric properties of the migraine screen questionnaire (MS-Q) using categorical data methods.

**Material and methods:**

A total of 343 students at Mizan-Tepi University, Ethiopia, age range = 18–35 years were selected by a simple random sampling method to participate in a cross-sectional study. The respondents completed the MS-Q, a semi-structured socio-demographic questionnaire, and a visual analog scale for attention (VAS-A).

**Results:**

The cumulative variance rule (> 40%), the Kaiser’s criteria (Eigenvalue> 1), the Scree test and, the parallel analysis (minimum rank) identified a 1-factor model for the MS-Q with the factor loadings in the range of 0.78 to 0.84. Fit indices favored a 1-factor model of the MS-Q as indicated by comparative fit index (0.993), weighted root mean square residual (0.048), root mean square error of approximation (0.067), the goodness of fit index (1.00), and non-normed fit index (0.987). The values of the Factor Determinacy Index (0.953), marginal reliability (0.909), H-latent (0.909), H-observed (0.727), explained common variance (0.906) and the mean item residual absolute loadings (0.225) further complimented finding of the 1-Factor model. McDonald’s Omega (0.903) suggested adequate internal consistency. Discriminative validity was supported by significantly higher scores for the total and all the MS-Q items except one among those with complaints of attention.

**Conclusion:**

The categorical methods support the psychometric validity of the MS-Q in the study population.

## Introduction

Migraine is one of the most common types of primary headaches [1]. Migraine is being recognized as a significant health problem affecting the quality of life [2]. During university life, students often report increased levels of stress, depression, anxiety [3], and irregular sleep, all of which are associated with migraines. Based on available data, migraine is on the rise in both general populations [4] as well as university students [5]. A recent systematic review of the prevalence of migraines in university students has reported a pooled prevalence of 16.1% among males and 21.7% among female students [6]. Therefore, it is pragmatic to have a brief, easy, and self-administered screening tool with psychometric validity for screening migraines in the student population.

Despite the significant disability caused, migraine continues to be an under-diagnosed condition [7]. Previous authors have suggested the use of standardized questionnaires for diagnostic screening [8] that would aid in the proper diagnosis and management of migraine. Several such instruments were developed in the past to assist primary care physicians in the screening of migraine [9–12]. A brief, reliable, and valid questionnaire will be helpful to the primary care physicians in the screening of migraine and decrease its under-diagnosis.

Migraine screen questionnaire (MS-Q) is a brief measure of migraine screening with favorable diagnostic validity, test-retest reliability, and internal consistency-as determined by the Cronbach’s alpha [13]. The MS-Q is based on the International Headache Society (IHS) criteria for the diagnosis of migraine and can be easily administered. The clinical usefulness of the MS-Q and its ability to detect a hidden migraine was also confirmed in a recent study [14].

The psychometric characterization of migraine and headache tools has been inclined towards an examination of test-re-test reliability, concurrent validity, and internal consistency [7, 9–13]. Given the value of the MS-Q as a potential and clinically useful screening tool, further investigation of its measurement properties including internal consistency, factorial validity, and discriminative validity, especially taking account of the categorical nature of the MS-Q item score is needed. Previous works did not consider categorical data assumptions for assessing internal consistency [7, 13]. Factorial validity assessment is essential to establish the relationship between item scores and examine the validity of the theoretical construct. Factor analysis inspects and determines the proper way of interpretation of items scores and address issues of multicollinearity, singularity, and redundancy of items [15]. Therefore, the present study examined the factorial validity, internal consistency, and discriminative validity of the MS-Q according to categorical data assumptions in university students.

## Material and methods

### Participants and study design

Participants in this cross-sectional study were university students recruited from the Mizan campus of the Mizan-Tepi University, Mizan-Aman, Bench Maji Zone, Southern Nations, Nationalities, and Peoples' Region, Ethiopia, using a simple random sampling method. Three hundred and forty–three students with an age range of 18–35 years completed this study. Students with memory problems were excluded, as this would lead to a compromised data quality. The Institutional Ethics Committee, College of Medicine and Health Sciences, Mizan-Tepi University approved the research. The guidelines of Good Clinical Practice and the norms of the World Medical Association (WMA) Declaration of Helsinki (DoH), and ethical principles for medical research involving human subjects were followed. Objectives and procedures of the study were explained to all participants, and written informed consent was obtained.

### Procedures and measurements

An interviewer-administered study questionnaire package was provided to all participants. The package included a migraine screen questionnaire (MS-Q), a semi-structured socio-demographic questionnaire, and a visual analog scale for attention (VAS-A). These questionnaires were administered in English, considering the participants' inconsistent proficiency levels for reading Amharic, the official language of Ethiopia. Moreover, the medium of instruction in the Ethiopian universities is English.

### Migraine screen questionnaire

The migraine screen questionnaire (MS-Q) is a five-item migraine screening questionnaire developed for use in clinical practice and research settings both in the general population and occupational medicine [13]. The questionnaire is based on the international headache society criteria (IHS) on migraine diagnosis [16]. Each of the five items in this structured questionnaire has a dichotomous response option of yes/no. A score of 0 is assigned for each “NO” response and of 1 for each “YES” response. The total score is 5, where a cut-off point of ≥4 was used to indicate a case of migraine [13].

### Visual analog scale for attention

The visual analog scale for attention (VAS-A) was used to evaluate the self-reported level of the problem in maintaining attention. A 100 mm horizontal line where ‘0’ indicated ‘never’, a middle score of ‘5’ denoted ‘hardly ever’ and ‘10’ indicated ‘yes definitely’ was placed next to the question, ‘Do you have difficulties in paying attention?’. The participants were instructed to mark on the line that they feel represents their perception about the problem in maintaining attention. Those with a 0–5 score on the VAS-A were categorized as having normal attention, and respondents with a score of 6–10 were categorized as having attention complaints.

### Socio-demographic questionnaire

This semi-structured socio-demographic questionnaire consisted of five-items; one open-ended and four close-ended. These items collected information regarding age, attendance level in the classes, grade at last examination, gender, and religion. Height in meters and weight in kilograms was measured separately to predict body mass index.

#### Statistical analysis

SPSS software (version 23; Chicago, IL, USA) and Factor 10.8.04 were used for data analysis. Participants’ characteristics descriptions were examined using mean, standard deviation, frequency, and percentage. Univariate descriptive statistics were analyzed using skewness and kurtosis. Spearman correlation between the MS-Q item and total score indicated homogeneity and item discrimination. Mardia’s skewness and Mardia’s kurtosis were used to assess multivariate distribution. The sample size adequacy and suitability of the MS-Q score for factor analysis were determined by Bartlett’s test of Sphericity, Determinant, Kaiser-Meyer-Olkin (KMO) Test of Sampling Adequacy (95% confidence interval), communality and inter-item tetra-choric correlations.

Tetra-choric correlations (estimated using bootstrap sampling) for inter-item scores of the MS-Q were used for factor analysis because these are dichotomous variables. Exploratory Factor analysis (EFA) was performed using robust diagonally weighted least squares (RDWLS) with Promin rotation. Kaiser’s criteria (Eigenvalue≥1), the Cumulative variance explained rule (> 40%), Scree test, and the robust parallel analysis based on minimum rank were employed as measures of factor retention. Multiple fit indices from different categories were employed according to recommended norms [17–19]. Discrepancy functions, such as robust mean and variance-adjusted χ^2^, χ^2^/df and weighted root mean square residual (WRMR), absolute fit index- the goodness of fit index (GFI), tests comparing target model with the null model like comparative fit index (CFI) and non-normed Fit Index (NNFI), and non-centrality indices like the root mean square error of approximation (RMSEA) were employed [17]. RMSEA (≤ .08), WRMR (≤ 0.05) and χ^2^/df (≤ 3) indicated acceptable and/or excellent fit [20, 21]. For CFI, NNFI, and GFI, a value greater than 0.95 implied an excellent fit [20, 21].

The quality and effectiveness of the explored factor structure of the MS-Q were assessed using Factor Determinacy Index (FDI) and marginal reliability. FDI is the correlation between factor score(s) and is employed to assess closeness between individual differences and true individual differences in the factor score [22]. A value of 0.9 of FDI is required for individual assessment [22]. Marginal reliability is square of the FDI; Brown and Croudace, 2015 emphasized its application as a measure of the reliability of the corresponding factor score [22]. The construct replicability measure, i.e., H-index [23] including H-latent and H-observed [23] were employed. A value of 0.7 indicated a reasonable level of construct replicability [24]. H-latent is a measure of correlations between the factor and the continuous latent response score that is supposed to underlie the observed categorical scores of the MS-Q items. H-observed is a measure of correlations between the factors and the observed item scores and is necessarily lower than the H-latent [23]. Explained common variance (ECV) was used to explore closeness to unidimensionality. ECV is the fraction of common variance that is attributed to the general factor with a value of 0.70–0.85, implying acceptability of unidimensionality [25]. Item Explained Common Variance (I-ECV) is the percent of item common variance that can be attributed to a factor. Items with an I-ECV value of 0.8 and above can be selected for a factor or a unidimensional construct [23]. Item residual absolute loadings (I-REAL< 0.3) was used to explore the departure from unidimensionality. It is a measure of the absolute loadings of the MS-Q item scores on the second factor of minimum rank factor analysis. MIREAL is the mean of such absolute loadings, a value above 0.3 indicates a departure from unidimensionality [23].

The internal consistency was assessed by the greatest lower bound to reliability and the McDonald’s Omega. Discriminative validity was assessed by the Mann Whitney U test.

## Results

### Participants’ characteristics and preliminary item analysis

Table 1 details the participants’ characteristics of enrolled university students. The majority of the participants (77.8%) were in the age group between 20 and 24 years, and 67% of them had a normal body mass index (BMI) with an average BMI of 21.2 ± 3.4 kg/m^2^ (Table 1). More than half of the participants (56.5%) were in good academic standing, with grades ranging from good to excellent (Table 1). About one-fifth (19.5%) of the students had migraine (Table 1). Univariate descriptive statistics, homogeneity, and item discrimination results were reported in Table 2. Two item scores had skewness more than 1.0, and the three items score had a kurtosis index above 1.0, suggesting the application of categorical data methods for the factor analysis and the use of McDonald’s omega for the internal consistency [26, 27]. As shown, all the MS-Q individual item scores were significantly associated with the total MS-Q score (r = 0.68 to 0.76, *p* < 0.01).

**Table 1:** Participant characteristics

Characteristics	Mean ± SD/frequency
Age (yr)	21.08 ± 1.93
15–19^a^	51(14.9)
20–24	267(77.8)
25–29	15(4.4)
30–34	–
35–39	1(0.3)
Unreported	9(2.6)
BMI (Kg/m^2^)	
Underweight	44(12.8)
Normal	230(67.1)
Over-weight	18(5.2)
Obese	8(2.3)
Unreported	43(12.5)
Gender	
Male	23.9(69.7)
Female	85(24.8)
Unreported	19(5.5)
Attendance (percentage)	
50–60	4(1.2)
61–70	2(0.6)
71–80	14(4.1)
81–90	33(9.6)
91–100	186(54.2)
Unreported	104(30.3)
Grade	
Satisfactory (2–2.49 number grade)	23(6.7)
Good (2.5–2.99 number grade)	57(16.6)
Very good (3–3.74 number grade)	96(28.0)
Excellent (3.75–4 number grade)	49(14.3)
Unreported	118(34.4)
Migraine (According to MS-Q)^b^	
Migraine	67(19.5)
No migraine	276(80.5)

*SD* Standard deviation

^a^All participating students were 18 or above years at the time of enrollment in the study

*BMI* Body mass index

*MS-Q* Migraine screen questionnaire

^b^ Cut-off (MS-Q total score) ≥4

**Table 2:** Univariate descriptive statistics, closeness to dimensionality measures, communality and factor loadings of the Migraine Screen-Questionnaire (MS-Q) scores in Ethiopian university students

Items of the MS-Q	Skewness	kurtosis	Item-total Correlations^a^	I-ECV	IREAL	Communality (h^2^)^b^	Factor loading^b^
Do you have frequent or intense headaches?	0.259	−1.930	.75^*^	0.913	0.247	0.707	0.798
Do your headaches usually last more than 4 h?	1.158	−0.660	.68^*^	0.885	0.303	0.797	0.835
Do you usually suffer from nausea when you have a headache?	1.289	−0.340	.65^*^	0.999	0.030	0.688	0.802
Does light or noise bother you when you have a headache?	0.454	−1.791	.76^*^	0.816	0.428	0.914	0.848
Does headache limit any of your physical or intellectual activities?	−0.012	−1.997	.76^*^	0.977	0.118	0.662	0.781

^*^p < 0.01

^a^ Spearman’s correlation coefficient

^b^ Diagonally weighted least squares for unrotated solution

*I-UniCo* Item Unidimensional Congruence, *I-ECV* Item Explained Common Variance

*I-REAL* Item Residual Absolute Loadings

### Factorial validity

#### Sample adequacy and sample suitability for factor analysis

The MS-Q scores in the studied university students fulfilled the conditions for the factor analysis. There were adequate linear combinations between the MS-Q item scores, as indicated by the results of Bartlett’s test of sphericity (< 0.001) [28]. No problems of multicollinearity and singularity were present in the MS-Q item scores, as suggested by the determinant score (0.282) [28]. There was a meritorious level of shared variance between the MS-Q item scores as implied by the Kaiser-Meyer-Olkin test of sampling adequacy (0.80) (Table 3) [28]. The inter-item tetra-choric correlations were in the range of 0.571 to 0.742, indicating moderate to strong correlations between MS-Q items (Table 4). This further supported the factorability of the MS-Q scores by substantiating evidence of the absence of both problems of multicollinearity and singularity [29]. All the item scores showed adequate communality conditions, i.e., all were above 0.4 for retention (Table 2) in the factor analysis [30].

**Table 3:** Multivariate descriptive, sample size adequacy, quality and effectiveness of factor score, construct replicability and reliability measures of the Migraine Screen-Questionnaire (MS-Q) scores in Ethiopian university students

Measures	Values
Multivariate descriptive	
Mardia’s skewness	Χ^2^(df = 35) = 271.190, *p* = 1.00
Mardia’s kurtosis	Χ^2^ = 0.513, *p* = 0.3038
Quality and effectiveness of factor score estimates^a^	
Factor Determinacy Index (FDI)	0.953
expected a posteriori marginal reliability	0.909
Construct replicability^a^	
H-Latent	0.909
H-Observed	0.727
Sample size adequacy	
Bartlett’s test of Sphericity	Χ^2^(df = 10) = 430.3, *p* < 0.001
Determinant	0.282
KMO (95% confidence interval)	0.804 (0.770–0.845)
Internal consistency	
Greatest Lower Bound to Reliability	0.932
McDonald’s Omega	0.903

Kaiser-Meyer-Olkin Test of Sampling Adequacy (KMO)

^a^ For the 1-Factor structure of the MS-Q

**Table 4:** Inter-item tetra-choric correlation matrix of the Migraine Screen-Questionnaire (MS-Q) scores in Ethiopian university students

Items of the MS-Q	Mean ± SD	MSQ-1	MSQ-2	MSQ-3	MSQ-4	MSQ-5
MSQ-1	0.44 ± 0.50					
MSQ-2	0.25 ± 0.43	0.742				
MSQ-3	0.23 ± 0.42	0.623	0.683			
MSQ-4	0.39 ± 0.49	0.613	0.631	0.708		
MSQ-5	0.51 ± 0.51	0.598	0.595	0.571	0.731	

*SD* Standard deviation

#### Exploratory factor analysis

Exploratory factor analysis results are presented in Table 5. Four tests were utilized to identify the number of factors (s) in EFA, i.e., the cumulative variance rule (> 40%), the Kaiser’s criteria (Eigenvalue> 1), the Scree test and the parallel analysis based on minimum rank, which is one of the robust measures of factor retention. As shown in Table 5, all the above said measures identified a 1-factor model for the MS-Q. The factor loadings of the MS-Q items ranged from 0.78 to 0.84 (Table 2).

**Table 5:** Summary of the factor extraction measures used in exploratory factor analysis of the Migraine Screen-Questionnaire (MS-Q) scores in Ethiopian university students

Number of Factors	Eigenvalue	Cumulative VA^b^	Above point of inflection on Scree plot	PA based on minimum rank			Decision to extract			
				Real-data VE^b^	Mean of random VE^b^	95th percentile of random VE^b^	Kaiser’s criteria (Eigenvalue≥1)	Cumulative VE rule (> 40%)	Scree test	PA 95th percentile of random VE > Real-data VE
1	3.599	71.982	Yes	88.8386^b^	59.6842	76.9187	^a^	^a^	^a^	^a^
2	0.521	10.420	No	9.2623	32.3088	44.6539	Χ	Χ	Χ	Χ
3	0.409	08.185	No				Χ	Χ	Χ	Χ

^a^ indicates extraction criteria fulfilled, Χ indicates otherwise

^b^ Values in percentage; *VE* Variance Explained, *PA* Parallel analysis

#### Fit indices

Except for a robust mean and variance-adjusted χ^2^ (df = 5) = 12.692, *p* = 0.027, all fit statistics provided adequate model fit to the data, i.e., the 1-factor model approximated the specified guidelines for CFI (0.993), GFI (1.00), WRMR (0.048), RMSEA (0.067 (0.00–0.111)) and NNFI (0.987). Furthermore, χ^2^/df was in the ideal range, i.e., 2.538 [19–21].

#### Quality and effectiveness of factor score estimates, construct replicability and measures of closeness to unidimensionality

The values of the FDI and marginal reliability were 0.953 and 0.909, respectively, for the one-factor structure of the MS-Q in the study population. H-latent and H-observed for the one-factor structure of the MS-Q were 0.909 and 0.727, respectively. ECV was 0.906, while I-ECV had a range of values between 0.816 and 0.999 for the 5-items of the MS-Q (Table 2). MIREAL was 0.225, while I-REAL had a range of values between 0.030 and 0.428 for the 5-items of the MS-Q (Table 2).

### Internal consistency and item discrimination

As shown in Table 3, adequate internal consistency was demonstrated by the greatest lower bound to reliability (0.932) [31] and the McDonald’s Omega (0.903) [32].

### Discriminative validity

The students with self-reported complaints of attention had significantly higher scores for all the MS-Q item scores (except MS-Q item-3) (*p* < .01) as well as the MS-Q total score (p < .01) than those with no complaints of attention (Table 6). A ROC curve analysis with the dichotomous variable of attention (No problem/Attention problem) and the MS-Q total score as test variable revealed an area under the curve of 0.66 with a 95% confidence interval of 0.58 to 0.74. At the cut-off score of 2.5 for the MS-Q total score, a sensitivity of 58.5% and a specificity of 69.7% was found to differentiate between those with attention problems and those without attention problems.

**Table 6:** Discriminative validity: Comparison of the Migraine screen questionnaire (MS-Q) scores between Ethiopian university students with attention complaints and no complaints of attention

Items of the MS-Q	Mean rank		*U*	*p*-value
	Attention complaints (*n* = 65)	No attention complaints (*n* = 238)		
MS-Q 1	174.90	145.75	6246.50	.006
MS-Q 2	170.77	146.87	6515.00	.010
MS-Q 3	161.78	149.33	7099.00	.162
MS-Q 4	184.23	143.20	5640.00	<.001
MS-Q 5	188.33	142.08	5373.50	<.001
MS-Q total score	189.57	141.74	5293.00	<.001

^*^Mean ± SD

## Discussion

This is the first paper to investigate the factorial validity, internal consistency, and discriminative validity of the original MS-Q using an appropriate analytical framework employing categorical data methods. A novel approach involving complementary measures was used to examine the quality and effectiveness of factor score estimates, construct replicability, and measures of closeness to unidimensionality. It is worth mentioning that this is the first psychometric examination of a migraine assessing tool in a previously uninvestigated population. Evidence showed that the unidimensional model of the MS-Q had adequate factorial validity, excellent internal consistency, strong internal homogeneity, and sufficient discriminative validity and item discrimination in the university students.

### Sample adequacy and sample suitability for factor analysis

The decision to conduct an EFA followed once all the indices of sample size adequacy measures indicated that the MS-Q scores were suitable for factor analysis as determined by KMO, Bartlett’s Test of Sphericity, the value of the determinant and moderate to strong inter-item tetra-choric correlations. All the five items were relevant for the construct validity of the MS-Q in the study population, as implied by the communality criteria [30].

### Exploratory factor analysis

In the EFA, all measures of factor extraction, including the robust measure of the parallel analysis based on the minimum rank [33], unanimously found a one-factor structure for the MS-Q. Furthermore, all five items in the MS-Q loaded on a single factor with factor loadings ranging from 0.78 to 0.84, which is higher than the minimum recommended factor loading score of 0.32 [34]. The range of factor loadings suggests that there was an excellent level of correlation between the MS-Q items and the factor score estimate [34].

### Model fit

The model fit indices analyses performed in the current study favored the one-factor structure of the MS-Q. Results of the majority of the model fit indices suggested that the one-factor model of the MS-Q adequately fits the data from our sample [19]. This is the first study examining the factorial validity and model fit of the MS-Q; hence a direct comparison with previously studied populations cannot be performed. Therefore, complementary measures like those assessing the quality and effectiveness of factor score estimates, construct replicability, and measures of closeness to unidimensionality were employed to establish findings of the factorial validity further. Factorial validity examinations have been generally under-utilized by studies investigating the psychometric validation of tools to screen migraine and headache. Though, factor analysis was employed to establish the multidimensional structure of the Headache Symptom Questionnaire-Revised in a pediatric population three decades ago [35]. Recently, Wang et al. 2017 employed factor analysis to determine the multidimensionality of a 27-item self-report of headache [36].

### Quality and effectiveness of factor score estimates, construct replicability and measures of closeness to unidimensionality

The values of the FDI for the MS-Q in the study population implied that there was an excellent level of comparability between the individual differences and true individual differences [25]. Therefore, the MS-Q met the condition for individual assessment of patients for screening migraine [25]. The reliability of the 1-Factor structure of the MS-Q was excellent as determined by the marginal reliability [22]. ECV further reinforced unidimensionality evidence found by EFA and model fit indices, because it was much higher than the minimum level for acceptance of unidimensionality requirements [25]. All the 5-items of the MS-Q fulfilled the criteria to load on the same factor as indicated by the I-ECV values above 0.8 [23]. Though there was a little concern about the departure from unidimensionality for the MS-Q item-4 because I-REAL for this item was above 0.3. However, the MIREAL, i.e., the average of all the I-REAL was well within the required limit, indicating no overall issues of departure from unidimensionality [23].

### Internal consistency and item discrimination

The present study reports a strong internal consistency of the MS-Q questionnaire, as evidenced by a McDonald’s omega value of 0.90. This indicates a strong relationship between each of the five MS-Q items. The original MS-Q development and evaluation study [13] reported similar internal consistency; however, the measure used in that study was Cronbach’s alpha coefficient. The present study used McDonald’s omega based on the fact that this a better alternative to Cronbach’s alpha when assessing the internal consistency of the scales with dichotomous responses [27].

Moreover, the choice of this estimate fulfilled the condition that the MS-Q items were found to measure a single latent construct where a one-factor model adequately represented the data [37]. All the item-total/Factor correlations were above 0.3; in fact, the lowest value was 0.65 (between MS-Q item-3 and the total score). This supports the conclusion that all the items of the MS-Q measured the same construct and, at the same time, showed sufficient item discrimination [38].

### Discriminative validity

Poor attention is associated with migraine; and headaches, both tension-type and primary headaches [39]. Inattention is 2.6 times higher in children and adolescents with headache [40]. Neurotransmitters like dopamine and noradrenaline are perhaps the pathophysiological connecting link between the attention deficit and migraine [41]. There is an inferential indication of overlap between neuro-anatomical cerebral circuits of headache and attention [42]. Therefore, significantly higher scores for the total and all the MS-Q items except one support a known group: discriminative validity of the MS-Q in the study population.

A brief account of the strengths and weaknesses of the present study is worth discussing here. The strengths include assessment of the factorial validity, internal consistency, internal homogeneity, known group: discriminative validity, and item discrimination using categorical data methods. Notably, scale development and evaluation research were criticized previously because of the inaccuracies in results reported due to the limited investigations in psychometric properties and validities of the scales [43]. One key aspect of this limited reporting is the factorial validation of the tools. This criticism applies to most migraine screening tools such as the MS-Q and ID migraine [9], as these tools have not undergone factorial validation using sound measures. The present study addresses this gap for migraine screening by reporting the results of factorial validation on the MS-Q. Another merit is the use of McDonald’s omega for assessing internal consistency following the requirements of the univariate distribution [27]. However, this study was limited by the narrow age group of the sample (from a university-level student population). The generalizability of the results may be limited to this age group from the socio-demographic group studied. It is recommended that in the future, multicentric studies with longitudinal design should be performed. Such studies may help investigate temporal and socio-demographic invariance of the factor structure of the MS-Q.

## Conclusions

Overall, the study findings provide further psychometric validation by providing evidence of adequate factorial validity, excellent internal consistency, strong internal homogeneity, and adequate discriminative validity and item discrimination in the study population. The findings of this study, along with those of previously published diagnostic accuracy studies in clinical populations, provide strong evidence for its use in screening migraine in both clinical as well as research settings.

## Data Availability

The datasets used and/or analyzed during the current study are available from the corresponding author on reasonable request.

## Abbreviations

MS-Q: Migraine screen questionnaire
EFA: Exploratory factor analysis
DWLS: Diagonally weighted least squares
KMO: Kaiser-Meyer-Olkin Test of Sampling Adequacy
CFI: Comparative Fit Index
WRMR: Weighted root mean square residual
RMSEA: Root mean square error of approximation
GFI: Goodness of fit index
NNFI: non-normed fit index
